# Serum fructosamine, serum glycated albumin and serum glycated β-lipoprotein in type 2 diabetes mellitus patients with and without microvascular complications

**DOI:** 10.1186/s40200-016-0276-0

**Published:** 2016-11-21

**Authors:** Tejaskumar R. Kalaria, Habibunnisha B. Sirajwala, Mukesh G. Gohel

**Affiliations:** 1 The Royal Wolverhampton NHS Trust, Wolverhampton, UK; 2 Government Medical College, Baroda, India; 3 Smt. NHL Municipal Medical College, Ahmedabad, India

**Keywords:** Type 2 diabetes mellitus, Microvascular complications, Fructosamine, Glycated albumin, Glycated LDL, NBT staining

## Abstract

**Background:**

Glycation of serum proteins has been proposed as an important mechanism of complications of diabetes but whether there are differences in glycation of different serum proteins and whether it has any correlation with development of microvascular complications has not been studied in depth. This study aimed to assess level of serum fructosamine, glycated albumin and glycated β-lipoprotein in type 2 diabetes mellitus patients with and without microvascular complications and to find out their correlation with diabetes complications.

**Methods:**

Case–control study involving 150 individuals at a tertiary care hospital in western India. Fifty participants were healthy controls (group 1), 50 were type 2 diabetes patients without any evident microvascular complication (group 2) and 50 were type 2 diabetes patients with one or more microvascular complications (group 3). Serum fructosamine, FBS, PP2BS and other biochemical parameters were measured. Glycated albumin and glycated β-lipoprotein were measured by agarose gel electrophoresis followed by NBT staining. Unpaired *t*-test was used to find out significance of difference between two groups and correlation coefficient to find out statistical correlation between two variables.

**Results:**

Type 2 diabetes patients with one or more microvascular complications had poor glycemic control as indicated by markers of short and mid-term glycemia. Differences between the groups for fructosamine, glycated albumin and glycated β-lipoprotein were significant (*p* < 0.001). Glycated albumin correlated with FBS, PP2BS and fructosamine in all diabetic patients (group 2 and 3) whereas glycated β-lipoprotein correlated with these parameters only in group 3 and it was markedly elevated in group 3.

**Conclusion:**

Serum glycated β-lipoprotein was disproportionately elevated compared to fructosamine and glycated albumin in diabetes patients with microvascular complications (group 3) and it correlated with rest of glycemic markers only in this group. Glycated β-lipoprotein might help in identifying diabetic individuals at high future risk of developing microvascular complications.

## Background

Hyperglycemia not only defines diabetes but it is also the cause of its most characteristic symptoms and long-term complications. Non-enzymatic glycation changes the structure and function of many soluble and insoluble proteins and it is probably the most important pathogenic mechanism amongst several proposed mechanisms. Because cells and their extracellular matrix share a dynamic relationship, modulation of matrix components by glycation leads to altered cell behaviour, including changes in cell spreading, phosphorylation of key intracellular signalling molecules, and expression of extracellular matrix proteins and their modulators. Accumulation of glycation products and the accompanying structural extracellular matrix modifications correlate with the development of complications of diabetes. These changes in tissue structure and function are slow and cumulative, producing a long time lag between the start of diabetes and the onset and progression of the complications [1]. Because the development of complications is linked to the accumulation of glycation adducts in tissue proteins, any analytical method that serves as an index of the extent of glycation can be used to guide therapy in diabetes. Amongst the various markers of glycemic control, glycated hemoglobin has now been established as a gold-standard long-term glycemic control index [2].

Glucose molecules are joined to protein molecules through glycation to form stable ketoamines, or fructosamines. It is a nonenzymatic mechanism involving a labile Schiff base intermediate and the Amadori rearrangement. The amount of fructosamine in serum is increased in diabetes mellitus owing to the abnormally high concentration of sugar in blood. The concentration of fructosamine in serum thus reflects the degree of glycaemic control attained by the diabetic patient over last 2–3 weeks and is useful in monitoring the effectiveness of therapy in diabetes over a period of several weeks, in a manner analogous to the determination of glycated hemoglobin [3]. Similar is the case with other glycated serum proteins depending on their half-lives, most widely studied and used of those is glycated albumin which is an indicator of glycaemic status over preceding 2–3 weeks. Due to 3–5 day circulating half-life of LDL, glycated LDL level reflects mean glycemia over the preceding week [4].

With availability of rapidly advancing information regarding molecular mechanisms, their inter-relations and their role in diseases, glycated serum proteins are coming to lime-light again. Glycated albumin, glycated LDL and glycated HDL have been found to be implicated in diabetic vascular diseases [5]. More and more studies are being conducted which assess these glycated proteins on molecular bases and, as a result, more and more new associations are being noticed. The results of the initial studies and also conclusions from other studies that rate of glycation of individual proteins shows biological variability from person to person at a same blood glucose level; have put glycated serum proteins beyond the markers of glycemia. Individual proteins are being measured and their associations are being sought for. This might lead to more and more markers coming from research setting to field level and some of these might even be specific for predicting a particular diabetic complication [5–7].

The study was undertaken to assess level of serum fructosamine, glycated albumin and glycated β-lipoprotein in type 2 diabetes mellitus patients with and without microvascular complications and to find out their correlation with the complications. Serum fructosamine was measured as an indicator of serum total glycated proteins and as a marker of short to medium term glycemic control.

## Methods

### Study design and subjects

This was a hospital based case–control study consisting of three groups and total 150 participants. Group 1 (control group, *n* = 50) consisted of randomly selected age and sex matched non-diabetic subjects. They were free from any ailment which could affect the parameters under study (no clinical history or investigative result showing involvement of any organ system). They were selected from medical and paramedical staff, attendants of patients and persons coming to hospital for fitness purpose. Group 2 (type 2 diabetes mellitus patients without any microvascular complications, *n* = 50) consisted of those with duration of diabetes 3 years or more, on life style modifications and oral anti-diabetic drugs and free from clinical or laboratory evidence of any microvascular complication of diabetes mellitus. Group 3 (type 2 diabetes mellitus patients with microvascular complications, *n* = 50) of those with duration of diabetes 3 years or more, on life style modifications, oral anti-diabetic drugs, insulin or combination of all three and diagnosed as having one or more microvascular complication of diabetes mellitus; either diabetic neuropathy (peripheral neuropathy diagnosed by 10 g monofilament and reduced vibration perception using 128 Hz tuning fork), diabetic nephropathy (urine albumin ≥30 μg/mg of creatinine with normal urine microscopy and not a known case of any other kidney disease) or diabetic retinopathy (diagnosed by direct ophthalmoscopy after mydriasis). Consecutive patients attending the medical outpatient department were enrolled in groups 2 and 3 unless they met any of the exclusion criterions.

Patients with type 1 diabetes mellitus, pregnant females and patients with diseases unrelated to diabetes which might significantly alter serum protein profile e.g. hepatic diseases, hematological malignancy, chronic infections and inflammations like tuberculosis, sarcoidosis, rheumatological diseases, infectious mononucleosis, AIDS were excluded from the study.

### Data collection and sample collection

Objectives of the study were explained to all subjects eligible for this study. Informed written consent of all the subjects was obtained for voluntary participation in study group, sample collection and for data utilisation for the purpose of publication. Data were recorded in a questionnaire designed for the study and it included socio-demographic data, presenting complains, detailed diabetes history including treatment and complications, history of other ailments, past, personal and family histories as well as findings of a thorough physical examination. For each subject, overnight fasting blood sample was collected in a fluoride vacutainer for FBS and in a plain vacutainer for other biochemical parameters. Urine sample was collected in universal container for urine creatinine and urine protein estimation. Post prandial (2 h) sample was collected in a fluoride vacutainer for PP2BS estimation. Serum and plasma were separated within an hour of collection and stored at 2 to 8 °C temperature till analyses were performed. Electrophoresis was performed within 5 days whereas all the other parameters were estimated on the same day.

### Sample analysis

FBS and PP2BS were estimated by Glucose Oxidase-Peroxidase (GOD-POD) enzymatic end point method. Serum fructosamine was estimated by NBT colorimetric kinetic assay. Serum Creatinine concentration was measured by modified Jaffe’s kinetic method. Serum total cholesterol was estimated by Cholesterol Oxidase-Peroxidase (CHOD-PAP) enzymatic end point method. Serum LDL cholesterol was measured by direct enzymatic method. Urinary creatinine was estimated by modified Jaffe’s kinetic method, urinary protein was measured by Pyrogallol red end point method and urinary protein/creatinine ratio was calculated. All biochemical investigations were performed on a fully automated analyzer.

Serum glycated β-lipoprotein was measured by agarose gel film electrophoresis at pH 8.6 followed by colour development with nitro-blue tetrazolium (NBT) by a modification of staining method adapted from Kunio Kobayashi et al. [8]. Dried and compressed electrophoresis plate was immersed for 20 h in glycated protein colour development reagent (5 mmol/l NBT in 50 mmol/l sodium carbonate buffer pH 10.3) at room temperature followed by two washes of stop solution (0.3% citric acid). NBT stained blue bands on the glycated protein film were scanned at 540 nm (Olympus glass filter plate) using a densitometric scanner (Nikon Super COOLSCAN 9000 ED). Image was analysed by free electrophoresis image analysis software GelAnalyzer 2010a. Proportions (%) of glycated albumin and glycated lipoprotein were represented as the percentage of total area under the scanned profile attributable to each peak. Concentration (in μmol/l) was calculated by multiplying percentage with serum fructosamine value. Staining in β region was considered to represent glycation of β-lipoproteins. Serum protein (stained with 0.5% acid blue 29 in 5% acetic acid) and lipoprotein (stained with 0.05% fat red 7B and 0.05% oil red O in methanol:water, 4:1) electrophoresis was performed simultaneously for all the samples to aid in quantitation by comparison of mobility of respective proteins. Run-to-run reproducibility of the assay of glycated albumin was 7.1% and for glycated β-lipoprotein was 7.7% when tested with a human serum sample with fructosamine value of 284 μmol/l (*n* = 10). The assay also performed satisfactorily when tested for linearity by using a very high fructosamine value (894 μmol/l) sample and its 1:1 to 1:8 dilutions.

### Statistical analysis

Data collected were recorded and analysed statistically to determine the significance of different parameters by MedCalc version 12.4. Statistical analysis was done by using unpaired *t*-test to find out significance of difference between two groups and correlation coefficient to find out statistical correlation between two variables and its significance. *p* value less than 0.05 was considered significant.

## Results

**Fig. 1:**
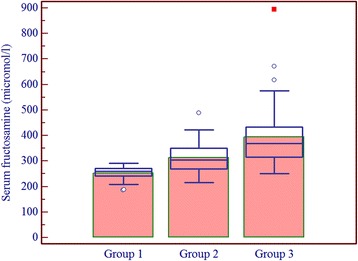
Box-and-Whisker distribution plot of serum fructosamine in study groups (*bars* indicate mean)

**Fig. 2:**
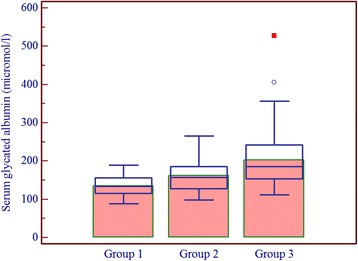
Box-and-Whisker distribution plot of serum glycated albumin in study groups (*bars* indicate mean)

**Fig. 3:**
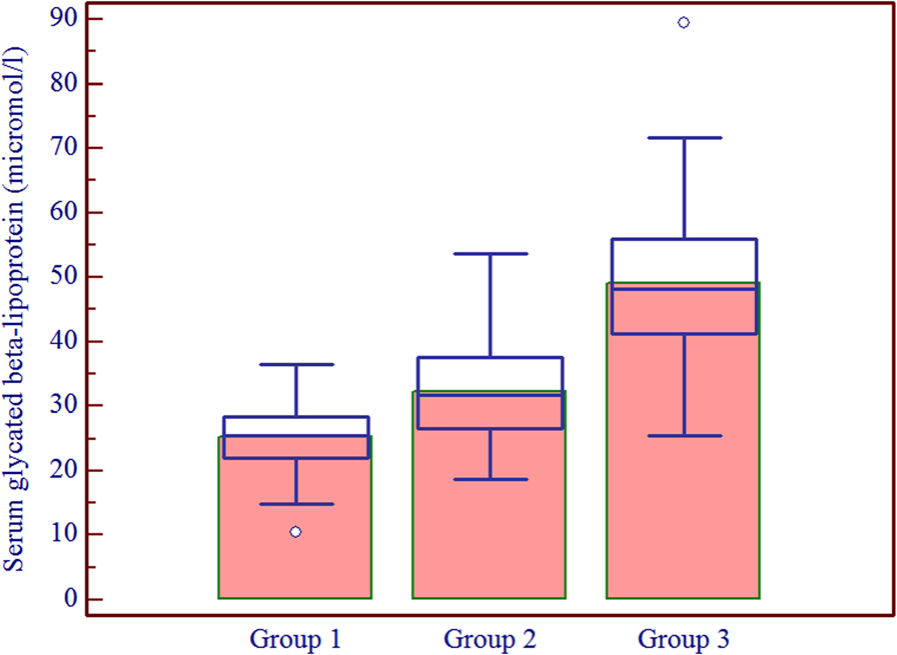
Box-and-Whisker distribution plot of serum glycated β-lipoprotein in study groups (*bars* indicate mean)

**Fig. 4:**
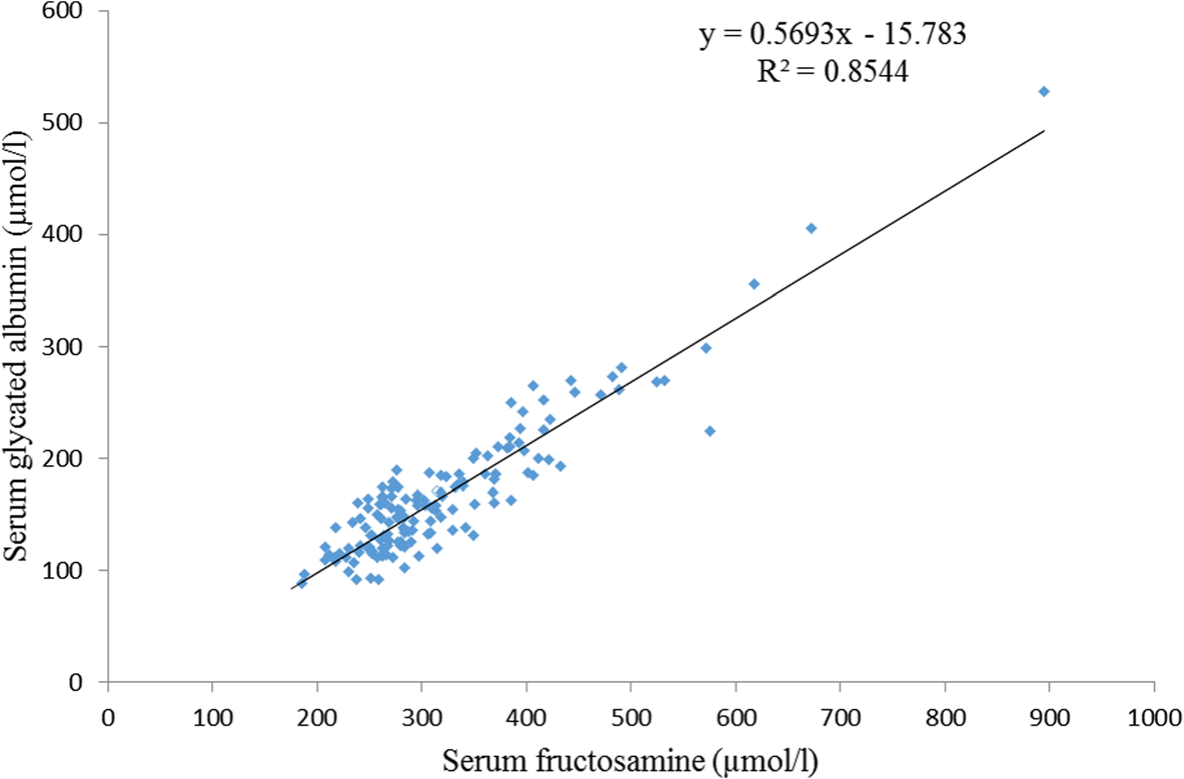
Correlation of serum fructosamine and glycated albumin (*n* = 150)

**Fig. 5:**
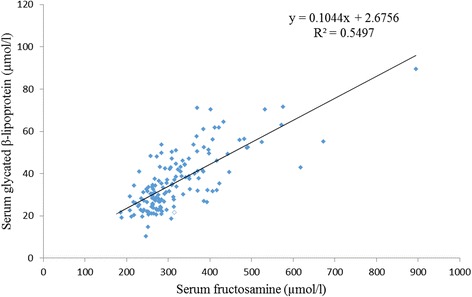
Correlation of serum fructosamine and glycated β-lipoprotein (*n* = 150)

**Fig. 6:**
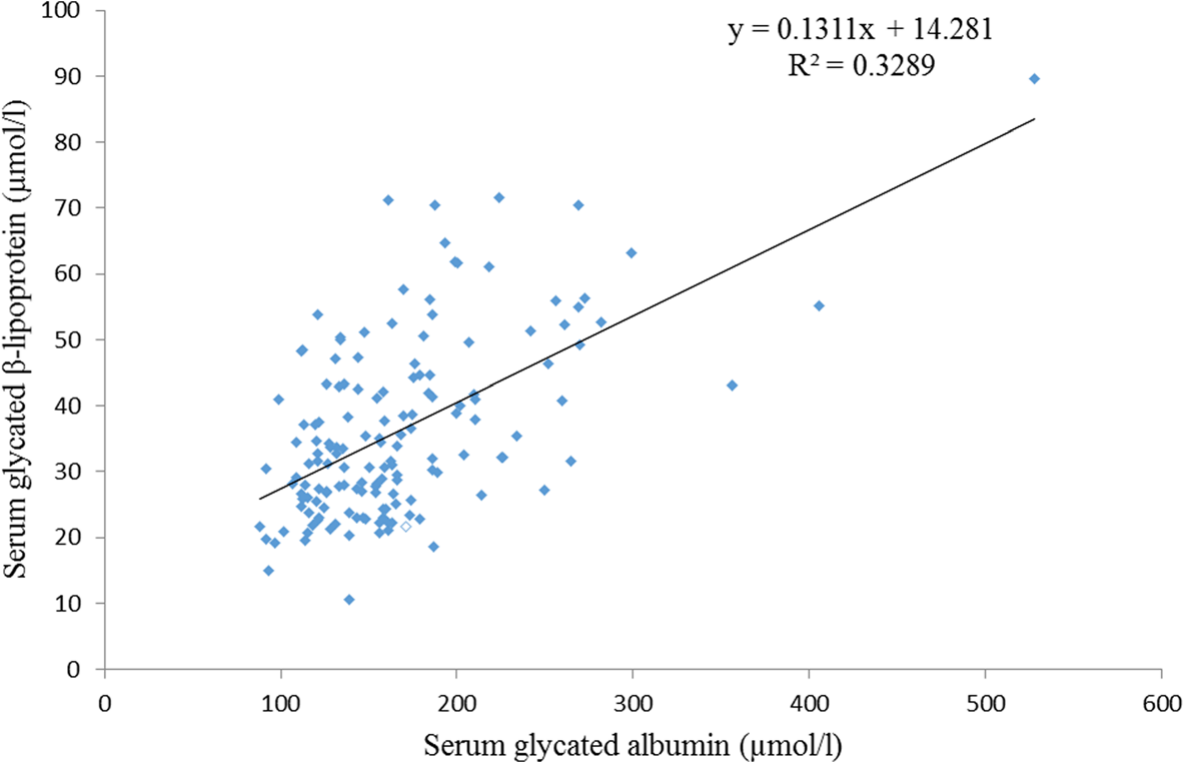
Correlation of glycated albumin and glycated β-lipoprotein (*n* = 150)

Patients having one or more microvascular complications were having poor glycemic control and large inter-individual variations as indicated by higher mean and larger SD values of glycemic indicators in group 3 (Table 1). Prevalence of fructosamine value higher than upper reference value (285 μmol/l) in group 1 was 2%, in group 2 was 60% and in group 3 was 92% and differences between these groups for serum fructosamine, glycated albumin as well as glycated β-lipoprotein were statistically significant (Table 2, Figs. 1, 2 and 3). Serum fructosamine and glycated albumin values correlated very well with both FBS and PP2BS in groups 2 and 3, whereas correlation was not observed in group 1 (Table 3). Glycated albumin showed excellent correlation with fructosamine across groups (*p* ≤ 0.0003 in all groups) and correlation coefficient r was >0.9 in groups 2 and 3 (Table 3). Glycated β-lipoprotein correlated well with FBS, PP2BS, fructosamine and glycated albumin only in group 3 (Table 3). Irrespective of the groups, serum glycated albumin showed excellent correlation with serum fructosamine whereas glycated β-lipoprotein level did not correlate well with either of fructosamine or glycated albumin, correlation with glycated albumin being particularly poor (Figs. 4, 5 and 6).

**Table 1:** Comparison of study groups

	Group 1	Group 2	Group 3
Number of participants	50	50	50
Sex (M/F)	58/42	54/46	48/52
Average age (years)	47 ± 10	53 ± 9	52 ± 9
Average duration of diabetes mellitus (in year)	-	6.3 ± 2.8	8.2 ± 3.8
FBS (mg/dl)	94 ± 11	129 ± 39	188 ± 86
PP2BS (mg/dl)	119 ± 11	184 ± 57	271 ± 109
Serum creatinine (mg/dl)	0.89 ± 0.20	0.96 ± 0.19	1.03 ± 0.29
Serum total protein (gm/dl)	6.8 ± 0.6	6.8 ± 0.5	6.7 ± 0.5
Serum albumin (gm/dl)	4.3 ± 0.3	4.4 ± 0.3	4.3 ± 0.3
Serum total cholesterol (mg/dl)	174 ± 35	177 ± 37	184 ± 43
Serum LDL cholesterol (mg/dl)	121 ± 29	117 ± 27	121 ± 27
Urinary protein/creatinine (mg/g)	11 ± 9	18 ± 8	140 ± 161
Serum fructosamine (μmol/l)	253 ± 24	313 ± 58	395 ± 119
Serum glycated albumin (μmol/l)	136 ± 26	162 ± 42	202 ± 78
Serum glycated β-lipoprotein (μmol/l)	25.2 ± 5.1	32.8 ± 7.8	50.4 ± 11.6

**Table 2:** Independent sample *t*-test: serum fructosamine, glycated albumin and glycated β-lipoprotein between study groups

Study groups	Fructosamine		Glycated β-Lipoprotein level		Glycated albumin	
	Test statistic t	Two tailed probability	Test statistic t	Two tailed probability	Test statistic t	Two tailed probability
1 and 2	6.761	*p* < 0.0001	5.719	*p* < 0.0001	3.751	*p* = 0.0003
2 and 3	4.388	*p* < 0.0001	8.937	*p* < 0.0001	3.235	*p* = 0.0018
1 and 3	8.316	*p* < 0.0001	14.043	*p* < 0.0001	5.728	*p* < 0.0001

**Table 3:** Correlation of study parameters

Correlation between parameters		Group 1	Group 2	Group 3
Serum fructosamine (μmol/l) and FBS (mg/dl)	*r*	0.0749	0.6446	0.7644
	*p*	0.6053	<0.0001	<0.0001
Serum fructosamine (μmol/l) and PP2BS (mg/dl)	*r*	−0.1174	0.5254	0.8142
	*p*	0.4168	0.0001	<0.0001
Serum glycated albumin (μmol/l) and FBS (mg/dl)	*r*	−0.1182	0.5085	0.7675
	*p*	0.9351	0.0002	<0.0001
Serum glycated albumin (μmol/l) and PP2BS (mg/dl)	*r*	−0.1785	0.3956	0.8023
	*p*	0.2149	0.0045	<0.0001
Serum glycated β-lipoprotein (μmol/l) and FBS (mg/dl)	*r*	0.0186	0.3702	0.5915
	*p*	0.8979	0.0081	<0.0001
Serum glycated β-lipoprotein (μmol/l) and PP2BS (mg/dl)	*r*	−0.1802	0.4013	0.6240
	*p*	0.2105	0.0039	<0.0001
Serum glycated Albumin (μmol/l) and fructosamine (μmol/l)	*r*	0.4896	0.9133	0.9491
	*p*	0.0003	<0.0001	<0.0001
Serum glycated β-lipoprotein (μmol/l) and fructosamine (μmol/l)	*r*	0.3626	0.3073	0.6661
	*p*	0.0097	0.0300	<0.0001
Serum glycated β-lipoprotein (μmol/l) and glycated albumin (μmol/l)	*r*	0.2152	0.1323	0.5625
	*p*	0.1295	0.3549	<0.0001

*r* = correlation coefficient, *p* = probability

Remarkably, glycated β-lipoprotein is specifically elevated in diabetes patients with microvascular complications (group 3) and it also shows large inter-individual variation as suggested by the SD value. The rise in mean glycated β-lipoprotein is disproportionate and not in line with rise in glycated albumin; as indicated by comparing the groups (Table 4). In group 3, as compared to group 1, there was 49% more glycated albumin whereas 100% more glycated β-lipoprotein.

**Table 4:** Change in parameters across groups in comparison to group 1 (considering group 1 values as 100%)

Parameter	Group 1	Group 2	Group 3
Mean fructosamine	100	124	156
Mean glycated albumin	100	119	149
Mean glycated β-lipoprotein	100	130	200
Glycated β-lipoprotein/glycated albumin	100	109	134

## Discussion

Findings in this study go hand in hand with the current establishments about serum fructosamine. Measurements of total glycated serum proteins and glycated serum albumin have been found in various studies, now over three decades, to correlate well with glycated hemoglobin and have been suggested as alternative methods for routine monitoring of glycemic control in patients with diabetes [9]. Additionally, there has been an increased interest to study these markers during recent past as they are now very well established as markers of intermediate term glycemia. There are many reports of intermediate term glycemic markers having their own role in addition to SMBG and the well-established ‘gold-standard’ glycemic marker HbA1c [4]. They have specific utility in glycemic monitoring of gestational diabetes mellitus and other conditions where HbA1c is not usable due to any reason [10]. These markers are immensely useful to adjust dosage of medication as they detect either improving or deteriorating glycemic status within a span of a week, much before the same changes in glycated Hb are evident (around four weeks) [3]. Fructosamine and glycated albumin are even being considered as diagnostic markers of pre-diabetes and diabetes [11, 12].

In contrary to previous beliefs, Kunio Kobayashi et al. demonstrated that fructosamine level in serum reflects not only glycation of albumin but also of lipoproteins. They also found that high concentration of glycated β-lipoprotein in diabetics is attributable not only to increase in the concentration of β-lipoprotein but also to accelerated glycation of lipoproteins in diabetics with hyperglycemia [8, 13]. Findings of the present study also suggest the same. T. J. Lyons et al. and C. M. Jack et al. using different methods found that, in diabetic patients, increased glycosylation of LDL occurs to an extent which correlates closely with other commonly used indices of glycemic control [14, 15]. In addition to its role in vascular complications, glycated LDL is also a short term glycemic marker, reflecting glycemia over past 1 week (3 to 5 day circulating half-life) [4, 16].

The most striking feature of this study was disproportionate elevation of glycated β-lipoprotein in diabetes patients with microvascular complications. The most plausible explanation for disproportionate elevation in glycated LDL is elevated small dense LDL in diabetes which is more susceptible to glycation than more buoyant LDL [17–20]. The capacity of glycated LDL for vascular damage is increased by its tendency to promote oxidation of the apolipoproteins themselves and the lipids in the particle core, yielding glycoxidized LDL. In many studies, glycoxidized LDL has been found to exaggerate cellular responses as previously mentioned with glycated albumin [6, 21, 22]. Precise mechanisms underlying why glycated beta-lipoprotein rise was disproportionate in diabetes patients with microvascular complication compared to those without them are yet to be understood.

Cellular effects resulting from glycation of LDL has been linked to both macro- and micro-vascular complications of diabetes. In vivo modified LDL from diabetic subjects as well as in vitro glycated LDL have been shown to enhance microvascular tone, which could contribute to impaired vascular reactivity in diabetes [23]. Studies have demonstrated that glycation diminishes the uptake and degradation of LDL by the high affinity LDL receptor, promotes uptake and metabolism by alternative pathways (e.g. monocyte-macrophages that give rise to foam cells) and decreases the rate of clearance of LDL in vivo [6]. Glycation also alters regulation of hydroxymethylyglutaryl-CoA reductase and acyl-CoA:cholesterol transferase activities, and accelerates free radical production and increases lipid peroxidation, which can enhance LDL atherogenicity through recognition and internalization of oxidized Apo B adducts by macrophage scavenger receptors and formation of foam cells. Macrophage uptake of glycated LDL is greater than that of control LDL [6, 21]. Glycated LDL has been shown to induce functional changes in various cell types including enhancement of chemotactic properties in monocytes, stimulating migration and proliferation of smooth muscle cells, and increasing platelet aggregation, nitric oxide production, and Ca^ + 2^-ATPase activity. Glycation of LDL increases its interaction with arterial proteoglycans and glycated LDL has also been found to trigger apoptosis of vascular endothelial cells by increasing the expression of apoptosis promoters [6, 24]. Glycation of LDL promotes oxidation of lipids and apolipoproteins and hence increases formation of glyoxidised LDL [25], which has been shown to enhance the cellular responses even further [6]. Endothelial dysfunction as a result of all these factors is closely linked with both micro- and macrovascular disease in diabetes [26]. Heavily oxidised and glycated LDL have been found to reduce expression of TIMP3 (tissue inhibitor of metalloproteinases 3) which might contribute to microvascular abnormalities in diabetic retinopathy [27]. Lyons et al. have demonstrated reduced cell viability in cultured retinal capillary cells after exposure to in vitro glycated vs native LDL [28]. Antibodies to oxidised LDL, glycated LDL and AGE modified LDL as well as the resulting immune complexes have pro-inflammatory effects and they increase foam cell formation and hence have been implicated in microvascular complications as well as atherosclerosis [29]. In DCCT/EDIC cohort (type 1 diabetes), higher levels of oxidised LDL and AGE-LDL in immune complexes were associated with higher odds of developing abnormal albuminuria [30].

## Conclusion

Diabetic patients having one or more microvascular complications had poor glycemic control. Serum fructosamine and glycated albumin showed excellent correlation with each other across study groups. Moreover, they correlated well with FBS and PP2BS in diabetic patients and thus remain as important markers of short to medium term glycemic control. Strikingly, glycated β-lipoprotein was markedly increased in type 2 diabetes patients with microvascular complications and the rise was disproportionate to that in glycated albumin. This indirectly indicates that glycated β-lipoprotein might help in identifying diabetic individuals at high risk of developing microvascular complication in future; and also lower LDL targets might be beneficial in diabetic individuals especially those having microvascular complications. Glycated β-lipoprotein correlated well with FBS, PP2BS, fructosamine and glycated albumin only in diabetes patients with one or more microvascular complication and not in the other two groups. As more and more such data becomes available and more studies of molecular mechanisms emerge, glycosylation of lipoproteins is likely to emerge as a very important mechanism of microvascular complication of diabetes.
